# How Can We Improve Teacher’s Work Engagement? Based on Chinese Experiences

**DOI:** 10.3389/fpsyg.2021.721450

**Published:** 2021-11-25

**Authors:** Danhui Zhang, Jingwen He, Dingmeng Fu

**Affiliations:** ^1^Collaborative Innovation Center of Assessment toward Basic Education Quality, Beijing Normal University, Beijing, China; ^2^Faculty of Education, Beijing Normal University, Beijing, China

**Keywords:** teacher autonomy, work engagement, China, basic psychological need, motivation

## Abstract

Drawing on Self-Determination Theory, the current study analysed the relationship between teachers’ perceived autonomy support and work engagement while it also explored the mediating effect of basic psychological need satisfaction and intrinsic motivation. The study investigated 520 elementary teachers in Beijing, and we found the following: (1) teachers in different groups reported diverse senses of perceived autonomy support, in that teachers with less teaching experience as well as those with a master’s degree have a higher score regarding the perceptions of teacher autonomy; and (2) teacher autonomy can affect work engagement not only in terms of the satisfaction of basic psychological needs but also by the chain of satisfaction of basic psychological needs and intrinsic motivation. Teachers with more autonomy support will have higher basic psychological need satisfaction and stronger teaching motivation, which will further enhance their work engagement.

## Introduction

Teachers’ work engagement not only affects their own professional development (Lyons, 2006) but also influences students’ physical and mental growth, as well as academic performance (Ruzek, 2012). Moreover, continuous school development depends on teachers’ engagement in work and their willingness to achieve school goals (Somech and Ron, 2007). A high degree of work engagement is generally connected with more commitment, better involvement, and increased productivity (Timms and Brough, 2013). Highly engaged teachers are usually satisfied with their jobs (Høigaard et al., 2012), and they are more likely to demonstrate organizational citizenship behaviours (OCB) and innovative behaviour (Konermann, 2012). Unfortunately, as teaching is one of the most stressful occupations (Skaalvik and Skaalvik, 2011), the problem of work engagement is universally recognized (Klassen et al., 2012a). Teachers with lower engagement often endure burnout, health problems (Hakanen et al., 2006), and severe turnover (Bal et al., 2013).

Historically, providing teachers with autonomy has been widely regarded as one of the most important approaches to solving teacher-related problems (Melenyzer, 1990), including low job satisfaction, burnout, and high turnover (Pearson and Hall, 1993; Davis and Wilson, 2000; Brunetti, 2001; Yao and Shen, 2007; Skaalvik and Skaalvik, 2014; Cai et al., 2018). There are two important but different rationales behind this theory. First, from the perspective of school management and administration, teacher autonomy is related to the feeling of providing teaching authority to those with the power to decide how to teach, without much direct supervision (Pearson and Hall, 1993). Such power could be used to unlock teachers’ potential for their creative and active involvement in work. Second, according to Self-Determination Theory (SDT), as professionals, teachers should be given greater autonomy and freedom; satisfying their basic psychological needs unlocks greater competency to positively influence their students (Pearson and Moomaw, 2006; Klassen et al., 2012b). There is rather robust literature on how autonomy could facilitate congruence between teachers’ perceptions on stress, job satisfaction, empowerment, and desire to remain in their current career (Pearson and Moomaw, 2005). For example, previous studies indicated that teachers’ perceived autonomy can positively predict their professionalism (Pearson and Moomaw, 2005), teaching commitment (Skaalvik and Skaalvik, 2014), and teaching self-efficacy (Lu et al., 2015). Some researchers also found that constraints on autonomy are related to greater frustration and anxiety among teachers (Dinham and Scott, 1996).

Although the significance of teacher autonomy has been thoroughly addressed, there are still several underexplored gaps in the literature. First, most of the explorations on autonomy support focus on the effect on students without such support. As evidenced by some empirical studies, including both experimental and longitudinal designed research, teachers’ autonomy support could prompt students’ learning engagement (Gregory et al., 2013; Stroet et al., 2013; Lam et al., 2014; Zhang et al., 2020). However, few studies provide empirical evidence and discuss how autonomy support influences teachers’ work engagement (Pelletier et al., 2002; Aelterman et al., 2014; Klaeijsen et al., 2018).

Second, most studies focus on teachers’ autonomy support from the perspective of empowerment (Hakanen et al., 2006; Field and Buitendach, 2012). Only a limited number of studies have attempted to explain why teacher autonomy might predict positive teaching outcomes from a psychological perspective (Skaalvik and Skaalvik, 2014). The research question needing further attention is: What is the potential psychological mechanism behind such relationships?

Third, although teacher autonomy has been thoroughly examined in other cultural contexts, especially in Western countries (Iyengar and Lepper, 1999), it is only now starting to receive more attention in the East, China included. Some cultural determinists have argued that autonomy support is less valued in Eastern cultures where collectivism is heavily encouraged (Jang et al., 2009) and the consensus is obedience to authority (Chirkov, 2011). Nevertheless, in China, this situation changed as the generation born after The Cultural Revolution became the major workforce. Influenced by market economy values, this generation embraces the freedom of choice and the pursuit of personal goals. Thus, it is worthwhile to explore the significance of autonomy support in Chinese context. Our research focuses specifically on school teachers for two reasons. First, the current education reformation set “providing autonomy support for students to foster their self-directed learning skills” as a major goal. As Ryan and Deci (2020) pointed out creating an autonomy-supportive learning environment conducive to motivating students intrinsically requires supporting teachers’ basic autonomy needs first. Second, Chinese school teachers are experiencing more challenges posed by both schools and parents, and, without exception, they are suffering from excessive workloads and serious burnout (Cai and Zhu, 2013; Hu et al., 2015). Traditional restrictive work environment is bound to make the situation worse. As a result, many Chinese schools begin to recognize the importance of granting teachers more autonomy.

In summary, the paucity of explanatory and empirical studies examining the relationship between teacher autonomy and work engagement from the perspective of psychology, and the difference between Chinese and Western contexts, provide a compelling rationale for the current study. In this present paper, we adopted a two-dimensional construct of teacher autonomy and aimed to investigate teacher autonomy in China, with an additional objective of explaining the mechanism underlying the relationship between teachers’ perceptions of autonomy support and their work engagement using the theoretical framework of SDT. This study proposes the following research questions:

1. Are there any differences among demographic variables such as gender, years of teaching experience, and academic qualifications in terms of perceived teacher autonomy support?
2. Can teacher autonomy affect work engagement by influencing basic psychological need satisfaction and teaching motivation?

## Theoretical Background

### Self-Determination Theory

Self-Determination Theory has been accepted as an approach for explaining the function mechanism between human beings’ psychological needs and their inner motivations, which in turn facilitates positive personal functioning (Ryan and Deci, 2017), including engagement (Ryan and Deci, 2000; Meyer and Gagne, 2008; Haivas et al., 2013). It sees human beings as active organisms with innate potential for psychological growth and development (Ryan and Deci, 2000). From the SDT perspective, autonomy support is hypothesized to meet individuals’ three basic psychological needs and thus fulfil individuals’ inner motivational resources as a prerequisite for facilitating high-quality engagement (Gagné and Deci, 2005; Reeve and Halusic, 2009). These relations were observed cross-culturally (Deci et al., 2001; Kaplan and Madjar, 2017). Based on such rationale, autonomy support is the initial drive and power and could facilitate the following positive changes. The satisfaction of the three basic needs (autonomy, competence, and relatedness) is the most direct outcome of autonomy support. Higher intrinsic motivation and engagement in turn would be influenced by the fulfilment of needs.

Research on enterprise management discovered that the autonomy of employees and their perception of autonomy support can predict not only their satisfaction with the three internal psychological needs, but also their performance and psychological adjustment (Baard et al., 2004). Furthermore, researchers have argued that teachers’ autonomy support for students could demonstrate an impact on students’ learning engagement by satisfying their need for autonomy and then providing intrinsic motivation (Garon-Carrier et al., 2016; Sparks et al., 2016; Yu et al., 2016; Orsini et al., 2018).

### Teacher Autonomy

According to Pearson and Hall (1993), teacher autonomy refers to a feeling of control in the work environment, particularly within the school context. This concept is composed of two dimensions: general teaching autonomy and curriculum autonomy. General autonomy concerns teachers’ general work discretion in school, which helps teachers to improve the capabilities of critical thinking and foster their creativity in teaching. Curriculum autonomy refers to instructional planning and implementation, such as selecting teaching activities and materials, regulating students’ learning, cultivating their learning motivation, and evaluating learning outcomes. It empowers teachers to have autonomy in teaching and learning decisions (Pearson and Hall, 1993; Pearson and Moomaw, 2006).

School-supported teacher autonomy plays an important role in forming teachers’ positive attitudes on working. With a higher degree of autonomy, teachers will perceive more joy and enthusiasm (Evertson and Weinstein, 2006), thus improving their decisions regarding continuing in the teaching profession (Pearson and Hall, 1993). More importantly, autonomy support can even predict teachers’ motivation to apply innovative pedagogies such as project-based learning (Lam et al., 2010), which is of great significance to their professional development. Unfortunately, most teachers experience more control than autonomy from school leaders (Pelletier et al., 2002). China is not an exception.

The centralized education system in China is quite different from that of the Western world. In such a system, schools are implicitly compelled to comply with the “authority” of the education administration, and teachers are directly supervised by school principals (Bao, 2008), resulting in the neglect of teacher autonomy in schooling and teaching (Su, 2007). Jia (2014) explained such systems from three perspectives. First, teachers are demanded to successfully undergo different types of evaluations and inspections conducted by the government, especially for compulsory education, with only one National Curriculum Standard for all schools established by the government. Teachers are required to follow detailed guidelines illustrated in the Standard. Second, given the hierarchically structured school system, principals usually do not value teachers’ autonomous competency and are more likely to foster conformity. Finally, under the pressure of standardized examinations, teachers take on more responsibilities to enable students pass the test; thus, teachers have less freedom in their procedures. In summary, increased curriculum control, the pressure of improving academic achievement, and more accountability to the school result in less teacher autonomy in the current Chinese context.

Tang (2014) investigated teacher autonomy from multiple perspectives: teachers’ willingness to develop autonomous teaching, their emotional feelings in autonomous teaching, making decisive choices by themselves in teaching, and autonomous teaching behaviours in real practice. Based on the results, although teachers showed strong willingness to develop autonomous teaching, they were in practice unable to actually make autonomous choices and decisions due to external influences, such as pressures and authority requirements. Moreover, teachers’ own teaching competency and confidence impede them from doing so, which resulted in a lower level of autonomous teaching behaviours.

### Teachers’ Three Basic Psychological Needs

As explained in SDT, each individual has three basic psychological needs (autonomy, competence, and relatedness). Regarding teachers, competence needs refer to how to finish a task or achieve a goal successfully. It is connected to a sense of efficacy (Krapp, 2005). In the school context, competence needs refer to teachers’ sense of achievement and recognition of their abilities in their daily teaching work. Autonomy focuses on choices and feelings. When the needs of autonomy are satisfied, an individual can better adjust his behaviour according to needs and available abilities, thus coordinating and prioritizing self-maintenance processes more effectively. Teachers’ autonomy need is the feeling that they can freely control the work they are engaged in. They can arrange the teaching work independently, and they can do so based on their own teaching ideas and design with the knowledge that what they are doing is respected and supported by school leaders and colleagues. Relatedness needs are focused on building a relationship of mutual respect and trust with others, thus giving a sense of belonging to a real or virtual group with common interests (Baumeister and Leary, 1995). Teachers’ relatedness needs refer to the perception of being accepted by the team, valued by leaders, and in harmony with colleagues. They have a strong sense of belonging in the team (Li, 2014).

According to SDT, teachers, as independent individuals with an innate potential for psychological growth and development, can recognize their own psychological needs and make free choices by accepting external environmental information (Ryan et al., 1997). Consequently, teaching initiative is determined by the degree to which the environment meets teachers’ three basic psychological needs (Cai et al., 2018). Many studies have confirmed that teacher autonomy has a significant positive impact on the three basic psychological needs of teachers (Deci et al., 2001; Roth et al., 2007; Klassen et al., 2012b; Korthagen and Evelein, 2016).

Some researchers have actually pointed out that satisfaction of basic needs could promote teachers’ interest-related motivation (Krapp, 2005; Wagner and French, 2010), professional development (Wagner and French, 2010), improvement of teaching expertise (Cai and Zhu, 2013; Li, 2014), and intention to change teaching strategies (Aelterman et al., 2016); satisfaction of basic needs is also negatively related to emotional exhaustion (Kaplan and Madjar, 2017). When competence, autonomy, and relatedness needs are satisfied, people will become more active at work (Anja et al., 2008).

### Teachers’ Intrinsic Motivation

Intrinsic motivation is another significant variable in the SDT (Ryan and Deci, 2000). Basic psychological need satisfaction is a necessary condition to support intrinsic motivation (Lam et al., 2010; Wagner and French, 2010; Klaeijsen et al., 2018). If the external environment can meet the three internal needs of competence, autonomy, and relatedness, the internalization and self-integration of external values will benefit, thus helping to initiate self-motivation. Conversely, such a scenario will reduce people’s intrinsic motivation and lead to adverse consequences (Chirkov et al., 2003; Baard et al., 2004).

Students’ intrinsic motivation in learning has always been a focus of both education and psychology research. There are few studies on “teaching motivation” (Han and Yin, 2014). Intrinsic teaching motivation reflects teachers’ subjective needs and value orientation in teaching, which plays an active role in influencing teaching behaviour. Specifically, intrinsic motivation refers to teachers’ thoughts and feelings concerning their own motivations for teaching (Roth et al., 2007). Teachers with a higher degree of intrinsic motivation would show stronger interests, more enthusiasm, and greater self-expectation in work (Zhong et al., 1999).

Studies have shown that teachers’ intrinsic motivation can influence their professional development (Wagner and French, 2010), innovative behaviour (Klaeijsen et al., 2018), and instructional methods (Robertson and Jones, 2013). Research has also demonstrated that teachers’ motivation can positively predict student engagement (Deci et al., 1996; Demir, 2011). Similarly, Robertson and Jones (2013) argue that if teachers perceive higher autonomy support from school, they tend to possess higher internal motivation, thus demonstrating better teaching methods.

### Teachers’ Work Engagement

Work engagement is explained as a positive emotional and cognitive working status characterized by vigour, dedication, and absorption (Schaufeli et al., 2002). Each of these three characteristics is perceived as a dimension for measuring the construct of work engagement. According to Hakanen et al. (2006), vigour is characterized by high levels of energy and mental resilience while working, the willingness to invest effort in one’s work, and persistence in the face of difficulties. Dedication is characterized by a sense of significance, enthusiasm, inspiration, pride, and challenge. Absorption is characterized by being fully concentrated and happily engrossed in one’s work, whereby time passes quickly, and one has difficulties detaching from work.

In the framework of SDT, work engagement also reflects the self-regulation of motivation. Higher motivation can bring higher levels of performance, sustainability, and creativity (Ryan and Deci, 2000). Studies in other fields have confirmed the significant relations between intrinsic motivation and work engagement. In an educational work setting, a teacher’s intrinsic motivation plays an essential role in the implication of innovative measures in education reform (Lam et al., 2010). Innovative teaching practices require teachers to devote more time and energy to their work. Especially for younger teachers, engagement was enhanced by intrinsic motives such as the opportunity for personal development, responsibility, and colleague support (Guglielmi et al., 2016).

Few empirical studies on teacher engagement have been conducted in China. Li (2015) surveyed 2,185 middle school teachers from the eastern, western, and middle regions of China. The findings showed 77.5% of the participants as full of vitality and actively engaged in their work. Teachers with less than 5years of teaching experience showed the greatest work engagement, while those with 16 to 25years of experience showed the lowest. Meanwhile, a higher educational background was related to greater work engagement. Teachers with a master’s degree scored significantly higher than those with a lower education degree (Li, 2015).

## Rationale For the Present Study

Although the psychological mechanism between empowering individuals with more autonomy and observing positive effects has been explained well in SDT, few studies have provided empirical evidence demonstrating such influences on teachers. In such a relationship, two important mediating variables are critical: basic psychological need satisfaction and intrinsic motivation. However, no study has explored how they simultaneously influence work performance.

Most studies have explored the mediation effects of basic psychological need satisfaction connecting teacher autonomy and work performance (Deci et al., 2001; Anja et al., 2008; Klassen et al., 2012b; Li, 2014; Klaeijsen et al., 2018). For example, Klassen et al. conducted three studies to explore the mediating role of need satisfaction on the relations of teacher autonomy and work-related outcomes such as work engagement, burnout, and emotional exhaustion. Their study confirmed that autonomy support from supervisors can enhance feelings of autonomy, competence, and relatedness satisfaction. More importantly, they highlighted that relatedness with students is the key psychological need associated with teaching engagement and emotions (Klassen et al., 2012b).

Klaeijsen et al. (2018) also discussed the relationship among satisfaction with basic psychological needs, intrinsic motivation, and innovative behaviours. They discovered that basic psychological need satisfaction can directly predict intrinsic motivation and innovative behaviour. As they explained, teachers with a higher degree of basic psychological need satisfaction are more confident in their ability to deal with changes in their work and are more inclined to actively seek independent development in teaching, share resources with others, accept new teaching ideas, and try new strategies in teaching (Klaeijsen et al., 2018).

In China, Li Min used the Chinese version of the Utrecht Work Engagement Scale (UWES) to study the relationship between work engagement of middle school teachers and basic psychological need satisfaction. It was found that the satisfaction of teachers’ basic psychological needs could effectively improve their work engagement, encourage them to be innovative, reduce fear of difficulties, promote dedication and self-devotion, and ultimately enhance the effectiveness of teaching (Li, 2014). In addition, the positive relationship between intrinsic motivation and work engagement was also confirmed among the Chinese teacher group (Li et al., 2015).

**Figure 1:**
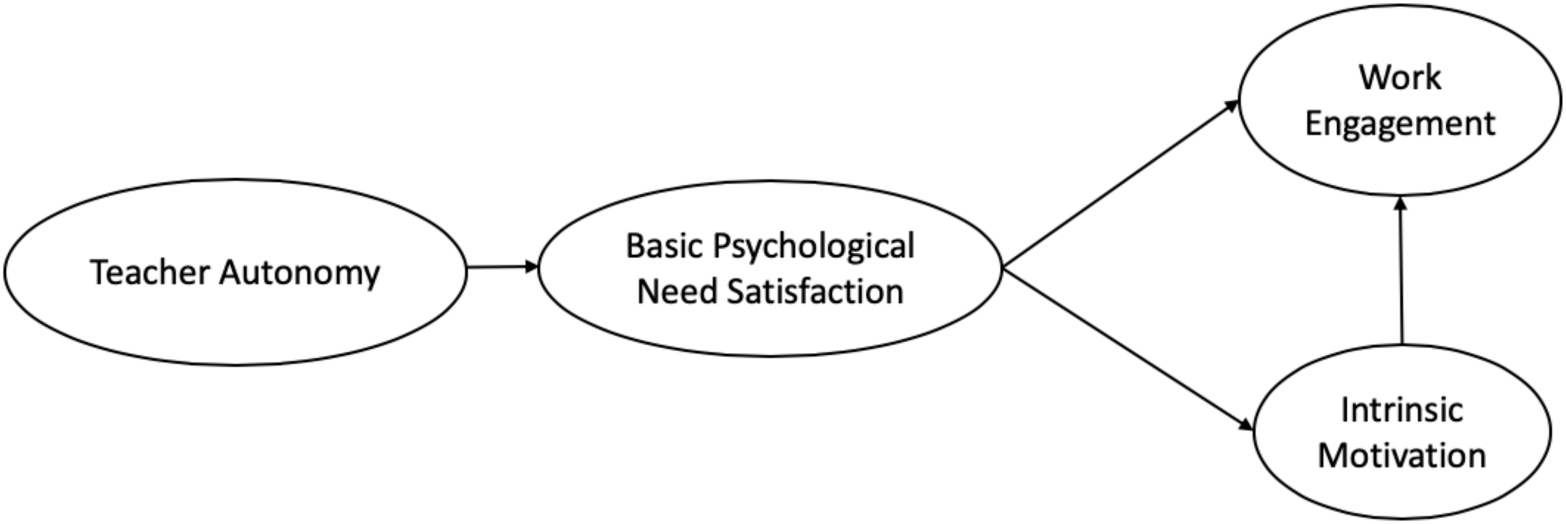
Self-determination theory model showing hypothesized relationships between teacher autonomy, satisfaction of basic needs (autonomy, competence, and relatedness), intrinsic motivation, and engagement.

Based on SDT, the present study attempts to test a conceptual model that states an intercorrelation between four constructs, in that teacher autonomy can promote teachers’ basic psychological need satisfaction, stimulate teachers’ teaching motivation, and ultimately enhance teachers’ work engagement. The conceptual model for our investigation is presented in Figure 1. It is based on both theoretical foundation and empirical evidence from previous studies.

## Materials and Methods

### Participants

The current study surveyed 520 teachers (446 women; 85.8%) with a mean age of 36.8years (SD=8.34) from 12 elementary schools in Beijing, China. The schools were first randomly sampled from schools of medium-size and average education quality in four administrative districts: HaiDian, ChaoYang, FengTai, and ShunYi. Then we contacted the school principals, introduced our research project and asked for their permission. Finally, we received consent from 12 schools in total. These schools are all ranked at the average level in Beijing.

The teachers have an average of 15.32years (SD=10.12) of teaching experience. All teachers were full-time certified teachers, of whom 12.7% obtained a master’s degree and 82.3% had a bachelor’s degree from a university. Of the teachers, 43.5 and 40.2% teachers teach Chinese and math, respectively. The rest of teachers are from the disciplines of English, physical education, arts, science, and music.

Participation in the study was entirely voluntary and written informed consent was required to conduct the questionnaires at each time. To protect the individual identification, all the participants were anonymous in this study. We did not disclose any personal information.

### Measurements

#### Teacher Autonomy

The Teaching Autonomy Scale compiled by Pearson and Hall in 1993 was adopted for this study (Pearson and Hall, 1993). The Chinese version of the scale has been adapted and revised by Chinese scholars (Yao and Shen, 2007). This 5-point Likert scale consists of two dimensions: curriculum autonomy and general teaching autonomy. Curriculum autonomy includes organising classroom activities, choosing teaching materials, and designing teaching processes (e.g., “What I teach in class is mainly my own decision”). General teaching autonomy refers to the establishment of routine teaching guidance standards and teaching work decisions (e.g., “I can exert creativity in teaching”). The scale was further revised. After excluding questions, seven items were left for measuring curriculum autonomy, and five were kept for measuring general autonomy. The revised scale showed good reliability and validity, *χ*^2^/df=3.145, RMSEA=0.064, CFI=0.946, TLI=0.930, and the internal consistency coefficient is 0.854.

#### Basic Psychological Needs

The Basic Need Satisfaction Scale compiled by Andrea, Marjan, and Rob Martens in 2018 was applied (Klaeijsen et al., 2018). The scale consists of 10 questions that are divided into three dimensions: autonomy, competence, and relatedness needs. The autonomy dimension contains 4 questions (e.g., “I am free to express my thoughts and opinions in my work”). The competency dimension contains 3 questions (e.g., “Most days, I feel a sense of accomplishment from work”). The relationship dimension contains 3 questions (e.g., “Colleagues at work will care about my feelings”). The scale displayed good reliability and validity, *χ*^2^/df=5.942, RMSEA=0.097, CFI=0.953, TLI=0.934, and the internal consistency coefficient is 0.913. All the items are on a 5-point-Likert scale ranging from 1 (strongly disagree) to 5 (strongly agree).

#### Teaching Intrinsic Motivation

The first “intrinsic motivation” part of the Teaching Motivation Scale compiled by Zhong, Shen, and Xin in 1999 was adopted in this study. The scale included 7 Likert-scaled items, with 1 indicating strongly disagree and 5 indicating strongly agree (e.g., “It’s a pleasure for me to engage in teaching work.”). This study showed that the scale also had good reliability and validity, *χ*^2^/df=7.106, RMSEA=0.108, CFI=0.979, TLI=0.963, and the internal consistency coefficient is 0.950.

#### Work Engagement

This paper adopted the Chinese version of the UWES (Schaufeli et al., 2002; Zhang and Gan, 2005). The scale is divided into three dimensions—vigour, dedication, and absorption—across sixteen items. The vigour dimension contains seven statements, such as “I feel energetic when I work.” The dedication dimension contains four statements, such as “I think what I am doing is very meaningful.” The absorption dimension contains 5 statements, for example, “When I work, my mind is full of work.” The result showed acceptable reliability and validity. The internal consistency coefficient is 0.936, *χ*^2^/df=4.201, RMSEA=0.078, CFI=0.948, TLI=0.936. This instrument employed a 5-point-Likert scale ranging from 1 (strongly disagree) to 5 (strongly agree).

### Data Analysis

Descriptive statistics, internal coefficients, and correlations among the study variables were computed using IBM SPSS Statistics 24.0. Possible associations between demographic variables (i.e., teacher gender, years of teaching experience, and educational level) and outcome variables (i.e., teacher autonomy, basic psychological need satisfaction, intrinsic motivation, and work engagement) were tested with an independent samples *t*-test and ANOVA.

In the next step, structural equation modelling was used to model associations between perceived autonomy support, basic psychological need satisfaction, intrinsic motivation, and work engagement. The relatively large sample size (*n*=520) supported the use of SEM with latent variables. Hence, the structural model was tested based on maximum likelihood estimation in Mplus. To evaluate the model fit, the absolute fitting index *χ*^2^/df, RMSEA and the relative fitting index CFI and TLI were selected. According to Hu and Bentler (1999), combined cut-off values close to 0.95 for CFI and TLI and close to 0.06 for RMSEA indicate a good fit.

## Results

### Descriptive Statistics

Table 1 presents information about background variables, including years of teaching experience, professional rank, and educational experience. The first record of degree refers to the full-time official diploma issued by the Ministry of Education of China before the teacher start working. Given that many teachers would continue the in-service education, we also asked about the highest degree they received.

**Table 1:** Distribution of gender, years of teaching experience, professional rank, and educational background (*n*=520).

Control variables		N	%
Gender	Male	74	14.2%
	Female	446	85.8%
Years of teaching experience	0–4	109	21.0%
	5–10	108	20.8%
	11–20	105	20.2%
	21–25	98	18.8%
	26–44	100	19.2%
Professional rank	No rank	37	7.1%
	Primary	438	84.2%
	Secondary	45	8.7%
First-record of degree	High school or below	205	39.4%
	College	60	11.5%
	Bachelor’s	225	43.3%
	Master’s	29	5.6%
	Ph.D.	1	0.2%
Highest degree	High school or below	3	0.6%
	Collage	21	4.0%
	Bachelor’s	428	82.3%
	Master’s	66	12.7%
	Ph.D.	2	0.4%

Teachers’ autonomy was conceptualized as a construct with two dimensions: curriculum autonomy and general teaching autonomy. Therefore, the total score of teachers’ perceived autonomy was composed by adding the domain scores of two dimensions. It was found that, on a continuous scale from 1 to 5, the average curriculum autonomy score of primary school teachers (Mean=3.11) is slightly lower than the mean general teaching autonomy score (Mean=3.87). The total score of teacher autonomy was 3.41.

The overall basic psychological need satisfaction was above the average level of 3 points, but there were distinct gaps in different dimensions. The relatedness need satisfaction score (Mean=4.09, SD=0.73) was the highest, while the autonomy need satisfaction score (Mean=3.37, SD=0.96) was the lowest of the three. The same trend was also observed in work engagement. Both the scores of dedication and absorption were higher (Mean=3.88) than those of vigour (Mean=3.39). The score of intrinsic motivation (Mean=3.95, SD=0.76) showed that teachers had relatively high intrinsic motivation. See Table 2 for detailed information.

**Table 2:** Descriptive analysis for main variables (*n*=520).

		M	SD
Teacher autonomy	Curriculum autonomy	3.11	0.88
	General teaching autonomy	3.83	0.60
	Total	3.41	0.69
Basic psychological need satisfaction	Competence	3.69	0.78
	Autonomy	3.37	0.96
	Relatedness	4.09	0.73
	Total	3.68	0.73
Teaching motivation	Intrinsic motivation	3.95	0.76
Work engagement	Vigour	3.39	0.79
	Dedication	3.88	0.83
	Absorption	3.88	0.70
	Total	3.67	0.69

Further Pearson bivariate correlation indicated a significant positive correlation among basic psychological need satisfaction, intrinsic motivation, work engagement, and perceived teacher autonomy support (See Table 3), which satisfies the preconditions for testing the mediation effect in structural equation modelling (Wen and Ye, 2014). With higher perceived teacher autonomy support, the degree of basic psychological need satisfaction, intrinsic motivation, and teacher’s engagement will also be enhanced.

**Table 3:** Correlation analysis for main variables (*n*=520).

S. No.		1	2	3	4
1.	Teacher autonomy	–			
2.	Basic psychological need satisfaction	0.439^***^	–		
3.	Teaching motivation	0.282^***^	0.604^***^	–	
4.	Work engagement	0.309^***^	0.623^***^	0.847^***^	–

^*^p<0.05; ^**^p<0.01; and

*** *p<0.001*.

### Analysis of Differences Among Groups

Independent samples *t*-test and ANOVA test indicated that teachers of different gender, professional rank, years of teaching experience, and educational background varied in their perceived autonomy support (see Table 4).

**Table 4:** Independent sample *t*-test and ANOVA results of teacher autonomy.

Demographic variables		N	Curriculum autonomy		General teaching autonomy		Total	
			Score	F/p	Score	F/p	Score	F/p
Gender	Male	74	3.30	1.968	3.81	−0.244	3.51	1.381
	Female	446	3.08	0.050^*^	3.83	0.087	3.39	0.168
Professional rank	No rank	37	3.44	3.090	3.98	2.161	3.67	2.822
	Primary	438	3.10	0.046^*^	3.80	0.116	3.39	0.060
	Secondary	45	2.99		3.92		3.38	
Years of teaching experience	0–4	109	3.50	8.193	3.93	2.819	3.68	6.560
	5–10	108	3.09	0.000^***^	3.72	0.025^*^	3.35	0.000^***^
	11–20	105	3.02		3.83		3.36	
	20–25	98	3.04		3.92		3.41	
	26–44	100	2.87		3.74		3.23	
First-record of degree	High school or less than	205	2.90	8.830	3.79	6.414	3.27	8.187
	College	60	3.01	0.000^***^	3.89	0.000^***^	3.38	0.000^***^
	Bachelor’s	225	3.28		3.85		3.52	
	Master’s	29	3.53		3.88		3.67	
Highest degree	Bachelor’s	428	3.08	−3.194	3.82	−0.844	3.39	−2.838
	Master’s	66	3.41	0.002^**^	3.87	0.401	3.60	0.005^**^

* *p<0.05*;

** *p<0.01; and*

*** *p<0.001*.

Gender difference was not significant in terms of general autonomy or total score. Men scored slightly higher than women only in the curriculum dimension. A similar pattern was observed for professional rank. Novice teachers with no rank scored significantly higher than teachers with secondary rank in terms of curriculum autonomy.

After grouping teachers’ years of teaching experience reasonably (see Table 1), it was found that teachers with 0–4years of teaching experience showed higher perceived autonomy support in both dimensions. In contrast, teachers with 26–44years of teaching experience scored the lowest in terms of curriculum autonomy.

The first and the highest academic qualifications of teachers were investigated respectively, as shown in Table 1. Because there were only two Ph.D.s, their data were deleted during the analysis. Teachers with a master’s degree scored significantly higher in both dimensions than those with a first degree of “high school or below.” Many teachers chose to attend continuing education to achieve higher degrees. Therefore, in terms of the highest degree, most of the teachers had already received a bachelor’s degree, accounting for 82.31% of the total surveyed teachers. An independent sample *t*-test showed that teachers with master’s degrees scored significantly higher on both general teaching autonomy and curriculum autonomy.

### Mediating Effect of Basic Psychological Need Satisfaction and Intrinsic Motivation in Teacher Autonomy and Work Engagement

**Figure 2:**
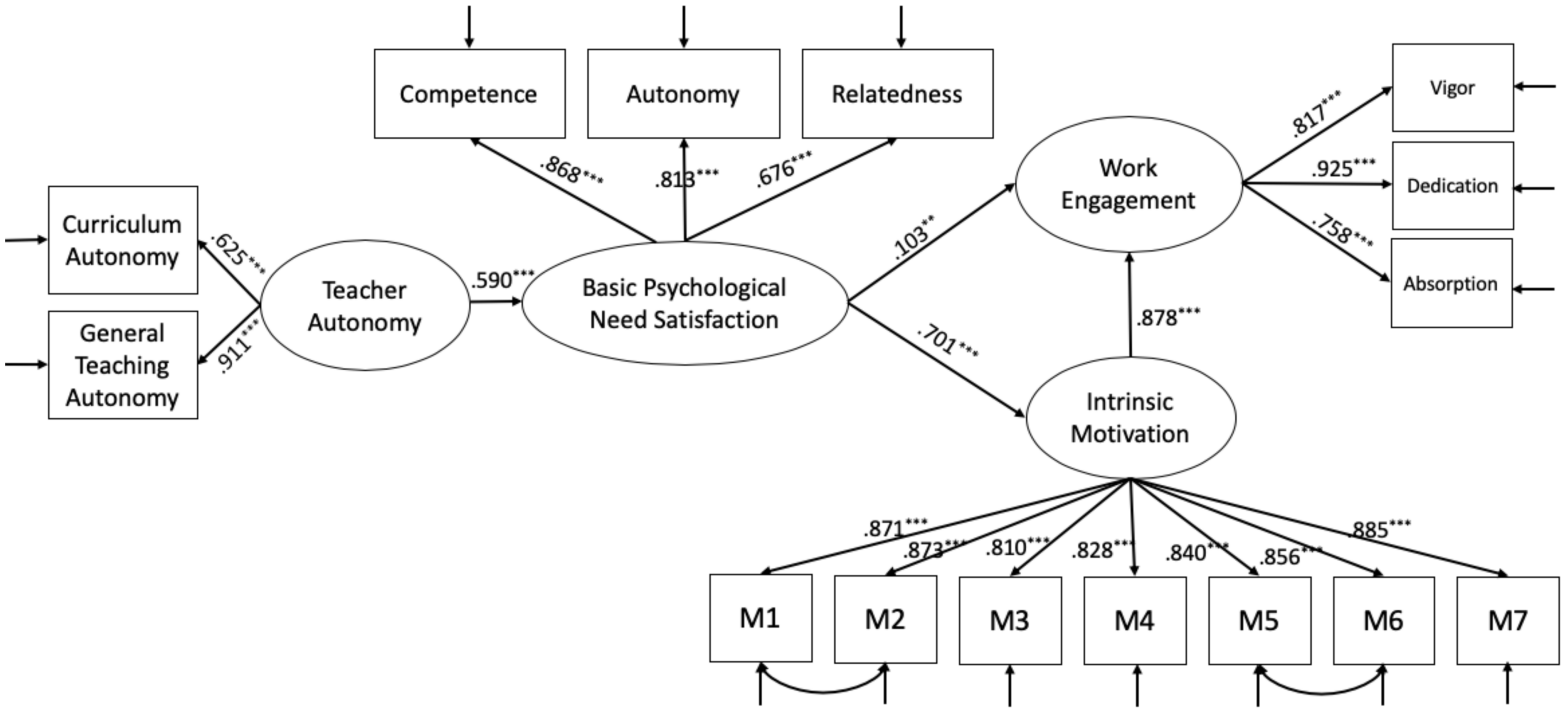
Model of teacher autonomy and work engagement. Model fit index: *χ*^2^/df=3.96; RMSEA=0.075; CFI=0.962 and TLI=0.952.

Based on correlation analysis, this study used a structural equation model to explore the mediating role of basic psychological need satisfaction and intrinsic motivation. A theoretical model predicting perceived autonomy support for work engagement was estimated using Mplus 7.0 software. It was expected that basic psychological need satisfaction and intrinsic motivation would partially mediate the relationship between need satisfaction and intrinsic motivation. The latent construct of perceived autonomy support was measured with two indices, namely, curriculum autonomy and general teaching autonomy. The latent construct of both basic psychological need satisfaction and work engagement was measured with three indices. The Mplus result showed that the proposed model fit reasonably well with the data, as shown in Figure 2.

The structural and measurement coefficients of the model are presented in Figure 2. All path coefficients were positive and significant (*p*<0.001). The path coefficient in the model showed that perceived teacher autonomy support had a positive predictive effect on teachers’ basic psychological need satisfaction (*p*<0.001). Need satisfaction had a positive effect on both teachers’ intrinsic motivation (*p*<0.001) and work engagement (*p*<0.01), while intrinsic motivation had a positive predictive effect on teachers’ work engagement (*p*<0.001).

The total indirect effect was 0.424, and a 95% confidence interval was calculated. The confidence interval of the mediation effect of basic psychological need and intrinsic motivation from teacher autonomy to work engagement was [0.229, 0.699]. The single mediation effect of basic psychological need was [0.003, 0.172]. The interval does not include 0, indicating that the mediation effect of the model was significant. The results showed that basic psychological need satisfaction and intrinsic motivation played an intermediary role between teacher autonomy and work engagement. Perceived autonomy support can affect work engagement from two paths: by only basic psychological needs and by both basic psychological needs and intrinsic motivation.

## Discussion

Given that work engagement could be viewed as a psychological state influenced by humans’ motivations, one possible way to enhance teachers’ work engagement is through a motivational approach (Li et al., 2015). According to SDT, the main benefits of perceived autonomy is that individuals’ basic psychological needs could be fulfilled, enabling teachers to be motivated intrinsically so that they could move further from “finishing their routine work” towards an active participation in the teaching activities in a positive way (Roth et al., 2007). Based on the survey of 520 primary school teachers from several schools in Beijing, the current study explored whether teacher autonomy can affect their work engagement through the mediating effect of basic psychological need satisfaction and intrinsic motivation.

The present study offers significant empirical evidence and implications for better understanding the application of SDT in different cultural context. First, this study was conducted in China, with a typical cultural context influenced by Collectivism, thus challenging the effectiveness of autonomy support (Chirkov, 2011). Second, under the Chinese centralized education system, schools follow standard guidance from the government, meaning that teachers experience more controlling rather than autonomous support from the school. Teacher autonomy was not recognized until recently. Third, we incorporated four important variables–autonomy, basic psychological need satisfaction, intrinsic motivation, and work engagement–in one integrated model, with the aim of examining whether the chained influences among variables examined in this model are consistent with the theoretical model in SDT as proposed by Deci et al. (2001) on the Chinese teacher group.

Several trends from the current study deserve further explanations. First, it was discovered that gender difference in total autonomy support was not significant, which was consistent with previous research (Pearson and Hall, 1993; Guo et al., 2014). However, we found that male teachers perceived more curriculum autonomy support than female teachers did. Based on further investigation, approximately 50% of these male teachers work in the fields of physical education, music, and art, which are not core courses, so they are less controlled by the Standards. Consequently, these teachers have more freedom in teaching, so they also perceive higher autonomy support. The second possible explanation is gender identity in the Chinese social context, in which women tend to be more obedient and willing to conform to authority, while men are more likely to be independent and insist on their own way. Therefore, male teachers are more likely to insist on their own approach to curriculum (Tang, 2014).

Second, novice teachers with a master’s degree showed higher perceived autonomy support, which differs from a previous study (Pearson and Hall, 1993; Tang, 2014). Chinese teachers attach great importance to educational background. Teachers with higher qualifications may also take leading roles, such as head teachers in school. Meanwhile, novice teachers with higher degrees are willing and capable to try different instructional strategies and have greater enthusiasm for their professional development. From the perspective of school management, school leaders generally recognize that teachers with master’s degrees have better academic and teaching skills training and will thus trust these teachers and give them more autonomy support.

The second finding deserving further explanation is the chained mediating effects of basic need satisfaction and intrinsic motivation connecting teacher autonomy and work engagement, which is established based on the theoretical framework of SDT. Initially, teacher autonomy showed a significant positive predictive effect on basic psychological need satisfaction, which in turn significantly predicts both intrinsic motivation and work engagement. Furthermore, intrinsic motivation also showed positive predictive influence on work engagement. Therefore, both basic psychological need satisfaction and intrinsic motivation play chained mediating roles between teacher autonomy and work engagement. Teachers perceive that more autonomy support will yield a higher sense of satisfaction of basic psychological needs, thus a stronger inner motivation for teaching and consequently a better work engagement.

The finding concerning the positive relationship between teacher autonomy, teachers’ basic psychological needs, and teacher engagement are consistent with the results of Klassen et al. (2012b). As illustrated in the SDT, granting teachers autonomy support is an efficient way to meet their inner psychological needs in autonomy, competency, and relatedness. The satisfaction of autonomy needs empowers teachers with more freedom to make their own decisions. Consequently, they have more desire to enact changes in behaviours, such as organizing and planning teaching activities, as well as their willingness to invest effort in teaching skill promotion. Therefore, autonomy need satisfaction will promote teachers’ vigour in teaching.

The satisfaction of competency needs not only lead to higher self-confidence but also strengthen teachers’ ambition to face new challenges. As teachers perceived more autonomy support from schools, they attached greater importance to their own professional development and became more goal oriented. Especially when teachers view the positive outcomes of autonomous teaching, such as rapid progress and growth of students, or when they receive positive feedback from school principals or other colleagues, their perception of accomplishment improves. As a result, they then devote themselves more to the current teaching work. Thus, competence need is related to work dedication.

Greater teacher autonomy enables teachers to have a stronger sense of belonging, so that their needs of relatedness will be fulfilled. The strong perception of relatedness indicates that the teachers are in a harmonious relationship with both colleague and students. Such a positive work atmosphere will in turn promote teachers’ willingness to take on extra tasks beyond their job description, such as volunteering to support school activities or helping other teachers (Runhaar et al., 2013). Teachers with higher satisfaction of relatedness needs will also receive more support from each other, and they are more likely to collaborate with each other, all of which will enhance the overall effectiveness of the school. As a result, teachers’ perception of relatedness will improve their work engagement.

Our findings regarding the mediation effects of intrinsic motivation connecting the basic psychological need satisfaction and work engagement also shed light on the importance of motivation in the teaching profession. Most previous research only focused on the direct effect of basic psychological need satisfaction on work engagement (Deci et al., 2001). According to SDT, the direct result of the enhancement of internal motivation will be reflected in individual behaviour change (Ryan and Deci, 2000). As they explained, motivation could be measured on a scale, with extrinsic motivation on one end and intrinsic motivation on the other (Ryan and Deci, 2000). For teachers with a higher degree of basic psychological need satisfaction, teaching is not just a job or a means of earning a living. Rather, they could achieve high self-fulfilment or accomplishment, as well as enjoyment and pleasure, from teaching. As a result, the intrinsic motivation can promote teachers’ enthusiasm for work, their sense of achievement in completing work, their courage to cope with challenges, and their degree of engagement.

## Practical Implications and Limitations

A school’s effectiveness is dependent on its teachers’ commitment and contribution. As such, teachers’ autonomy in school merits increased attention. Granting teachers more autonomy has not only been found to have paramount positive influences on teachers themselves but also on their students. Feinberg et al. (2005) found that respecting and supporting teachers’ autonomy can internalize their value of supporting students’ autonomy as well as making them more willing to learn various autonomous support strategies. After participating in the programme for 2years, teachers’ controlling behaviours decreased significantly, and student reports show that teachers’ pro-social behaviour in the classroom increased significantly (Feinberg et al., 2005). Therefore, teachers under greater control from the school tend to put more pressure and controlling strategies on their students. On the other hand, teachers who benefit from receiving more autonomy support from the school are more likely to recognize the value of inner psychological drive and try different approaches to motivate students intrinsically. For example, they might change from directly telling students the conclusions to giving them more time and opportunities to explore independently. They may respect the students as independent and autonomous learners, fostering students’ self-regulated learning strategies in the classroom.

How can we give teachers more autonomy support? In current school management, the most serious problem is that principals often forego establishing a supportive work environment, attaching more importance to the training of instructional strategies. Nevertheless, without the basis of a supportive, autonomous atmosphere, it is difficult for teachers to exert teaching methodologies. Jia proposed that it is necessary to guarantee teachers’ autonomy in several ways such as respecting the inherent demands of their professional autonomy; providing more autonomous space for teaching through democratic management; improving teaching skills through providing practical trainings; and strengthening teachers’ responsibility in autonomous teaching (Jia, 2014). These strategies work together to create a positive atmosphere, encouraging teachers to exert their own initiative and stay innovative in their teaching.

Two methodological limitations need to be considered. First, the sample was obtained from 12 elementary schools in Beijing. We recognize that generalizability will be an issue and recommend that future research involve a larger number of schools and teachers. The second limitation is that the current study only provided correlational results; such data might not provide implications inferring causality.

## Data Availability Statement

The raw data supporting the conclusions of this article will be made available by the authors, without undue reservation.

## Ethics Statement

The studies involving human participants were reviewed and approved by the Ethics Committee of the Collaborative Innovation Center of Assessment for Basic Education Quality, Beijing Normal University. The patients/participants provided their written informed consent to participate in this study.

## Author Contributions

DZ: research design and paper writing. JH: data collection and analysis. DF: design questionnaires. All authors contributed to the article and approved the submitted version.

## Funding

This research was funded by the 2019 (MOE) Ministry of Education in China Project of Humanities and Social Sciences (19YJC190026).

## Conflict of Interest

The authors declare that the research was conducted in the absence of any commercial or financial relationships that could be construed as a potential conflict of interest.

## Publisher’s Note

All claims expressed in this article are solely those of the authors and do not necessarily represent those of their affiliated organizations, or those of the publisher, the editors and the reviewers. Any product that may be evaluated in this article, or claim that may be made by its manufacturer, is not guaranteed or endorsed by the publisher.
